# Nuts and bolts of lung ultrasound: utility, scanning techniques, protocols, and findings in common pathologies

**DOI:** 10.1186/s13054-024-05102-y

**Published:** 2024-10-07

**Authors:** Michael Beshara, Edward A. Bittner, Alberto Goffi, Lorenzo Berra, Marvin G. Chang

**Affiliations:** 1 Department of Anesthesia, Critical Care, and Pain Medicine, Massachusetts General Hospital, Harvard Medical School, 55 Fruit Street, White 437, Boston, MA USA; 2 Department of Anesthesia, Critical Care and Pain Medicine, University of Kansas Medical Center, Kansas City, KS USA; 3 Interdepartmental Division of Critical Care Medicine, University of Toronto, Toronto, ON Canada; 4 Department of Medicine, Division of Respirology (Critical Care), University Health Network, Toronto, ON Canada; 5 Department of Medicine, University of Toronto, Toronto, ON Canada

**Keywords:** Point of care ultrasound, Lung ultrasound, Critical care ultrasound, POCUS, LUS, Ultrasound, Anesthesia, Pulmonary, Critical care, Mainstem intubation, Pneumothorax, Atelectasis, Pneumonia, Aspiration, COPD exacerbation, Cardiogenic pulmonary edema, ARDS, Pleural effusion, Ultrasound training, Ultrasound competency, POCUS training, POCUS competency, Lung ultrasound training

## Abstract

Point of Care ultrasound (POCUS) of the lungs, also known as lung ultrasound (LUS), has emerged as a technique that allows for the diagnosis of many respiratory pathologies with greater accuracy and speed compared to conventional techniques such as chest x-ray and auscultation. The goal of this narrative review is to provide a simple and practical approach to LUS for critical care, pulmonary, and anesthesia providers, as well as respiratory therapists and other health care providers to be able to implement this technique into their clinical practice. In this review, we will discuss the basic physics of LUS, provide a hands-on scanning technique, describe LUS findings seen in normal and pathological conditions (such as mainstem intubation, pneumothorax, atelectasis, pneumonia, aspiration, COPD exacerbation, cardiogenic pulmonary edema, ARDS, and pleural effusion) and also review the training necessary to achieve competence in LUS.

**Supplementary Information:**

The online version contains supplementary material available at 10.1186/s13054-024-05102-y.

## Introduction

Point of Care ultrasound (POCUS) of the lungs, also known as lung ultrasound (LUS), has emerged as a simple, non-invasive, and real time technique that allows for the diagnosis of many respiratory pathologies [1–6]. LUS can also be utilized to guide and assess the response to therapeutic interventions affecting the pulmonary system such as delivery of fluids, administration of diuretics, endotracheal intubation, ventilator management, and chest tube placements [7–12]. Health care providers may utilize LUS to diagnose common pulmonary pathologies and optimize their patients’ care in the same way they utilize their stethoscopes [13–15]. The goal of this review is to provide a simple and practical approach to LUS for critical care, pulmonary and anesthesia providers, as well as respiratory therapists and other health care providers to be able to implement this technique into their clinical practice. To achieve this, this review is structured to provide a basic overview of the physics of lung ultrasound, discuss the hands-on scanning technique, probe selection, describe LUS findings in normal and pathological conditions (such as mainstem intubation, pneumothorax, atelectasis, pneumonia, aspiration, COPD exacerbation, cardiogenic pulmonary edema, ARDS, and pleural effusion), and review the training necessary to achieve competence in LUS.

## Physics of lung ultrasound

Understanding the basic physics behind LUS is essential for optimization and interpretation of imaging techniques. LUS is heavily dependent on the interpretation of ultrasound artifacts as will be discussed in this article [16].

Air has been considered the enemy of ultrasound waves [17–19]. At the soft tissue and air interface, the majority of ultrasound waves are reflected because of an acoustic impedance mismatch. This is the reason gel is necessary when performing body surface imaging [11, 17]. Because of this, it was previously thought that the use of ultrasound in lung imaging would not be of any value because air in the lungs would cause a significant reflection of ultrasound waves to prevent imaging of the lung parenchyma [20]. While this may be true, the use of LUS doesn’t exclusively depend on the visualization of the lung parenchyma itself but rather on the detection of different ultrasound artifacts with unique signatures that allows the diagnosis of different lung pathologies [16, 17]. Bone also prevents ultrasound propagation and result in dropout artifacts [21, 22].This precludes imaging of structures that lie underneath the ribs. The attenuating effects of lung parenchymal air and the ribs therefore limit visualization to the pleura in the intercostal spaces between the ribs and thus the diagnosis of various respiratory pathologies depend heavily on the ability to recognize different ultrasound artifacts [23].

On LUS, the parietal pleura (outer pleural layer attached to the chest wall) and visceral pleura (inner pleural layer covering the lungs) appear as a hyperechoic horizontal line (Supplemental Fig. 1) referred to as the pleural line. The pleural line may represent the real pleura, or an artifact caused by ultrasound beam reflection between alveolar air and soft tissue, the exact cause is still debatable [19]. This pleural line moves with respiration and this movement is called “lung sliding” [11, 19]. Supplemental Video 1 shows common signs seen with normal LUS.

Cardiac oscillations are also sometimes visualized as low amplitude vertical oscillations at the pleural line, these oscillations result from the transmission of cardiac contractions and are referred to as the “lung pulse”. Lung pulse is a normal ultrasound finding and its presence implies an intact pleural interface and rules out pneumothorax (Supplemental Video 1) [7, 24–26].

Deep to the pleural line, and at regular intervals appear horizontal hyperechoic lines referred to as “A-lines” (Supplemental Fig. 1). These A-lines are a type of reverberation artifacts created by repetitive reflection back and forth of the ultrasound waves between two strong reflectors. This gets interpreted by the ultrasound machine as occurring at multiples of the distance between the probe and the pleural line. As seen in Supplemental Fig. 1, A-lines occur at equidistance intervals of the ultrasound probe to the strong reflector at the pleural line [11, 19, 27].

**Fig. 1:**
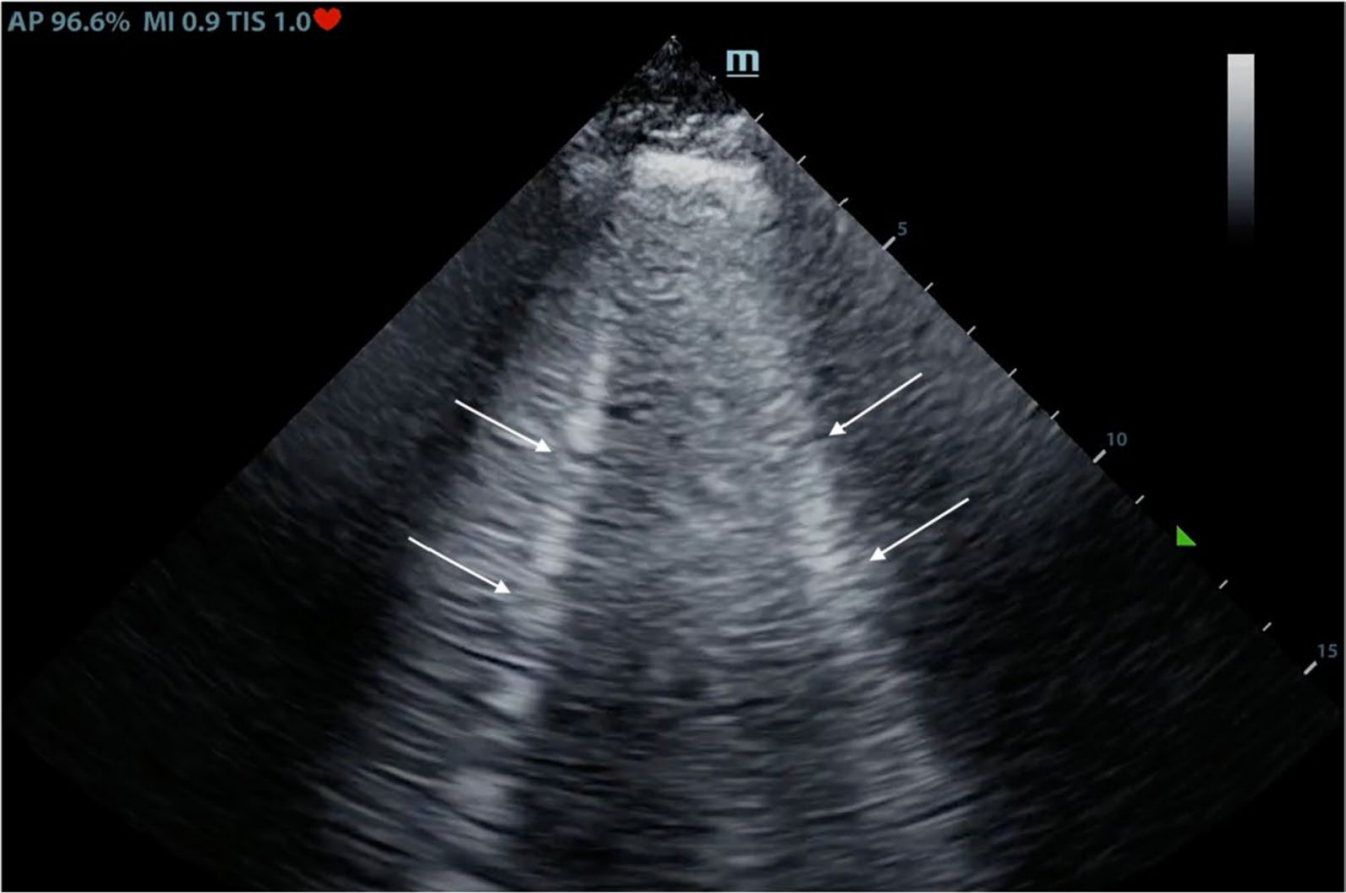
B-lines (white arrows) which are vertical hyperechoic lines that begin at the pleural line and extend all the way down to the bottom of the ultrasound screen

Another LUS artifact is known as “B-lines”. B-lines are vertical hyperechoic lines that begin at the pleural line and extend all the way down to the bottom of the ultrasound screen (Fig. 1). These “B-lines" should be differentiated from short vertical artifacts which extend for a short distance beyond the pleural line and are not considered B-lines (Supplemental Fig. 2) [28]. B-lines also represent a type of reverberation artifact often referred to as “comet tails” or “lung rockets”. This occurs when the ultrasound waves get trapped between two closely spaced reflectors (such as fibrous tissue and alveoli with any type of fluid such as pus, blood, water, and inflammation that is surrounded by air in the setting of pulmonary edema, pneumonia, ARDS, and connective lung diseases) and then reflected back to the transducer resulting in B line [28–32].

It is important to note that LUS doesn’t solely rely on the analysis of artifacts (A-lines and B-lines). In the absence of normally aerated lung, LUS is often times able to visualize pathology associated with the lung parenchymal and pleural space (e.g. consolidation, atelectasis, and pleural effusions) [28]. This will be discussed in more details in the coming sections of this article.

## Scanning technique and probe selection

A variety of ultrasound probes are useful for LUS, the linear, curvilinear, and phased array probes [33, 34]. Linear probes are high-frequency probes and are ideal for visualizing the pleural line and pathology that may affect the pleura (e.g., pneumothorax). The phased array and curvilinear probe are lower frequency probes and are best suited for the evaluation of deeper structures as in the case of pulmonary edema or consolidation [19, 33]. Compared to the phased array probe, the use of curvilinear probes may be associated with a higher interpretation accuracy especially in novice users trying to interpret pleural pathologies [35] and may also be associated with fewer number of detected B-lines [36]. The significance of these findings and its effect on interpretation accuracy of LUS need to be further explored in large randomized controlled studies.

We recommend placing the probe on the chest wall to allow the identification of landmarks including the ribs, subcutaneous tissue and the pleural line. The probe should then be tilted until the ultrasound beam is directed perpendicular to the pleura [37]. This will result in the appearance of 2 ribs with the pleural line visualized in between and this positioning provides the characteristic “bat sign” in which the upper and lower ribs resemble the wings of the bat with acoustic shadowing and the pleural line outlining the back of the bat (Supplemental Fig. 3) [7, 24, 25, 28]. This allows structures to be readily identified when analyzing still and dynamic images.

When performing LUS, the focus should be adjusted to be at the level of the pleural line. The gain should be reduced to allow visualization of the hyperechoic pleural line, A and B lines; however, when anatomically imaging the lung parenchyma, as in consolidated lung, the ultrasound gain will often need to be increased [33, 38, 39].

Most LUS examinations are initially performed with patients in the supine position. To examine the dorsal region of the lower lobes, patients may need to be positioned in the lateral decubitus position. The dorsal segments of the upper lobes are usually not visualized with LUS as they are located behind the scapula [40]. When evaluating for pleural effusions, a seated or semi-seated position is recommended to optimize visualization of the pleural fluid at the level of the costophrenic angle as fluid tend to accumulate in the most dependent portions of the chest.

From a practical point of view, scanning each lung at three points on each side as used in the modified Bedside Lung Ultrasound in Emergency (BLUE) Protocol (described in detail in the examination protocols section below) is usually sufficient to make a quick diagnosis in patients with acute respiratory failure [7, 25, 41]. However, scanning protocols differ in the number of the recommended scanning points and can be 6 points or more on each side [42, 43] which may be impractical to perform for every patient given the already increased workload of health care practitioners. In the original (BLUE) protocol published in 2008, scanning was performed at 3 zones on each side (anterior, lateral and posterolateral chest walls) and each zone was further divided into upper and lower halves resulting in 6 scanning points on each side [44]. In the 2012 consensus conference on point of care lung ultrasound, scanning at 4 lung areas on each side was recommended for a complete 8 zone LUS examination [11]. More simplified scanning techniques have been also described to look for signs of pulmonary congestion during stress echocardiogaphy or weaning from mechanical ventilation [45, 46].

## The normal exam

A normal LUS is characterized by the presence of lung sliding seen in 2-dimensional (2D) imaging [7, 11, 23, 25, 33, 40, 47]. With M-mode imaging, which represents the interrogation of a single vertical line within a 2D image, a normal lung sliding is seen as “seashore sign” (Supplemental Fig. 4) [48]. This sign results from the normal movement of the visceral pleura which creates a fuzzy or “sandy beach” image under the subcutaneous tissue and the parietal pleural layer reminiscent of a sea shore [7, 40, 44, 49, 50]. M-mode examination is sometimes useful when it is difficult to acquire a good 2D image of the pleural line showing lung sliding. Visualization of lung sliding on 2D ultrasound or a “seashore sign” on M-mode may be especially helpful in the setting of cardiac arrest to confirm endotracheal tube (ETT) placement as end-tidal CO2 may not be reliable in such a setting [6, 51].

Normal LUS exam is also characterized by the presence of lung pulse (Supplemental Fig. 5) and horizontal A-lines which are caused by reverberation artifact of the ultrasound beam as discussed before [7, 11, 19, 25, 40, 44, 49]. Isolated vertical B-lines (Fig. 1) and short vertical artifacts (also referred to as Z-lines) (Supplemental Fig. 2) are also commonly seen in normal lungs [28]. Usually, they are very few in number [24, 40, 52–54].

## LUS in different respiratory pathologies

Table 1 details the LUS findings found in a number of pathologies such as pneumothorax, mainstem intubation, atelectasis, pulmonary edema, pneumonia, pleural effusion, ARDS, and lung contusions. We also describe the LUS findings in these various pathologies in greater detail below.

**Table 1:** Summary of common LUS signs in different respiratory pathologies

Lung Pathologies	Common LUS findings
Pneumothorax	**No Lung sliding**, A-lines present, **No vertical artifacts**, **No lung pulse, ± Lung point**
Mainstem Intubation	**No Lung sliding**, A-lines present, B-lines may be present, **lung pulse present**
Atelectasis	No lung sliding, **B-lines present**, Positive Lung pulse, **Static Air bronchogram, Pulsatile flow likely absent on Color Doppler**
Pulmonary Edema	Positive lung sliding, **Bilateral pathologic B-lines ( ≥3/intercostal space)**, Positive lung pulse
Pneumonia	**Lung hepatization,** pathologic B-lines (mostly bilateral in viral and unilateral in bacterial pneumonia), **Irregular pleural line (Shred sign), Dynamic air bronchogram, Pulsatile flow likely present on Color Doppler**
Pleural Effusion	**Anechoic area between parietal and visceral pleura, Spine sign, Sinusoidal sign**, Plankton sign (in complex effusion)
ARDS	**Pathologic B-lines with spared areas and irregular distribution**, **Lung hepatization**, Dynamic air bronchogram, Loss of lung sliding, pleural line irregularities
Lung Contusion	**Pathologic B-lines**, Lung hepatization

### Pneumothorax

LUS in the hands of an experienced clinician can detect a pneumothorax with a much higher sensitivity and specificity compared to a portable chest x-ray [40, 55]. It also offers the advantage of being faster enabling the timely management of this critical diagnosis particularly in trauma cases where its application has been extensively described in the literature.

Since air tends to move to the non-dependent portion of the chest, LUS examination for pneumothorax should be performed with the patient is in the supine position, so the most non-dependent portion will be the anterior chest at the 2^nd^–4th intercostal spaces at the mid-clavicular line [40]. The presence of air between the visceral and parietal pleura will lead to the reflection of the ultrasound beam. As a result, the visceral pleura will not be visualized. Additionally, there is a loss of movement of visceral pleura against the parietal pleural resulting in loss of lung sliding [7, 11, 25, 44]. However, it is important to understand that lung sliding is also absent in other lung pathologies, including mainstem intubation, pulmonary adhesions, atelectasis, and with apnea [44, 49].

**Fig. 2:**
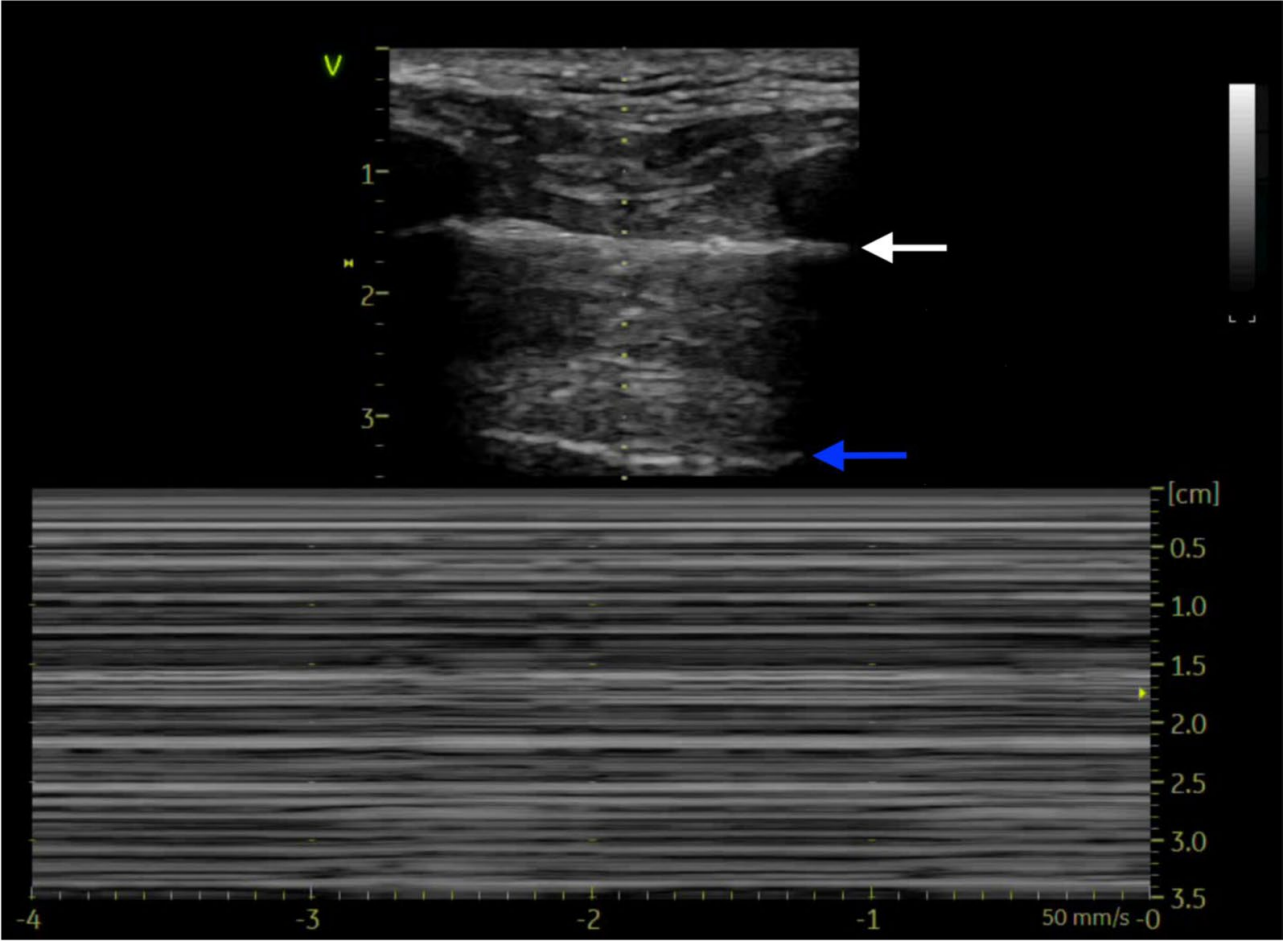
Top panel is a 2D image of pneumothorax depicting the pleural line (white arrow) and A-line (blue arrow) with no B-lines. Bottom panel is an M-mode image in pneumothorax showing stratosphere or Barcode sign

In pneumothorax, the absence of lung sliding will be associated with the presence of A-lines and the absence of lung pulse and B-lines or other vertical artifacts originating from the pleural line [11, 40, 49]. In M-mode, a pneumothorax is characterized by the “barcode sign” or “stratosphere sign” which is a series of interrupted white and black lines caused by the absence of lung sliding and lung pulse (Fig. 2) [49, 56, 57].

In a partial pneumothorax, the transition between the normal lung pattern and the pneumothorax is seen on 2D and M-mode imaging as a “lung point” (Supplemental Fig. 6). The presence of a lung point is highly specific for pneumothorax [7, 40, 44, 49, 58] however, such transition will not be seen in a complete pneumothorax. It is also essential to exercise caution when interpreting this sign, as other conditions like bullous disease, lung contusion, pleural thickening and adhesions can generate an ultrasound pattern similar to the “lung point” [59, 60]. It is also worth mentioning that the physiological pleural sliding on the heart can resemble the “lung point”, potentially resulting in a false diagnosis of pneumothorax or overlooking a tiny pneumothorax when air is located in the left paracardiac region [61].

**Fig. 3:**
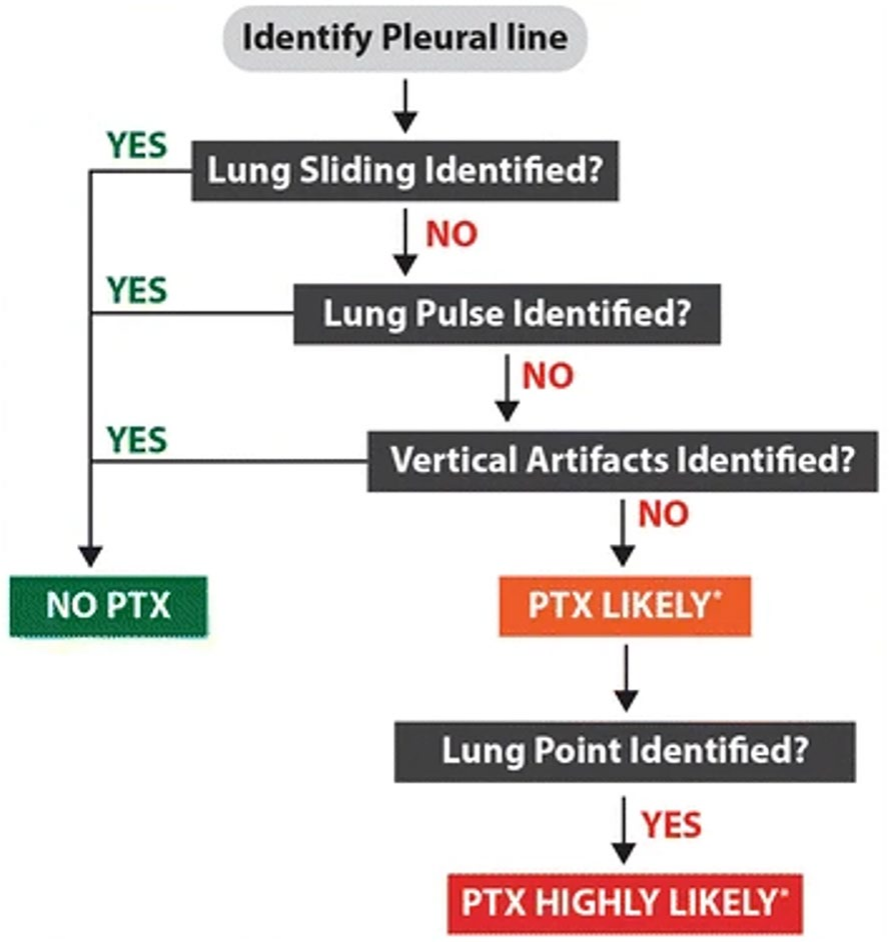
Simple Algorithm for diagnosis of Pneumothorax. Reproduced with permission from Dr. Goffi [62]

Figure 3 shows a simple algorithm for the diagnosis of pneumothorax from Goffi et al [62]. Supplemental Video 2 also shows ultrasound signs commonly seen in pneumothorax.

It should be also noted that in the case of subcutaneous emphysema, one will also not see lung sliding because the ultrasound beams will not travel through air in the subcutaneous tissue. Vertical hyperechoic lines known as E-lines may be seen in subcutaneous emphysema and sometimes confused with B-lines; however, E-lines don’t arise from the pleural line and they don’t move synchronously with respiration as in B-lines (Supplemental Fig. 7) [63]. The “bat sign” is also not visualized in the case of subcutaneous emphysema.

### Mainstem intubation

With a mainstem intubation there will be an absence of lung sliding in the non-ventilated lung. This occurs more commonly in the left lung, given the greater occurrence of right mainstem intubation because of the more vertical takeoff of the right bronchus compared to the left [64]. LUS was found to be superior to auscultation in detection of main stem intubation [65]. A lung pulse may be present in the setting of a mainstem intubation as there is no barrier between the visceral and parietal pleura that prevents transmission of cardiac movement to the parietal pleura.

### Atelectasis

A characteristic LUS finding with atelectasis are “air bronchograms” (Supplemental Fig. 8). Air bronchograms results from trapping of air within the bronchial tree of the lung tissue resulting in hyperechoic circles. With atelectasis, the air bronchograms are static and do not move with respiration [7, 41, 66, 67]. A lung pulse and B lines may be present in atelectasis. When atelectasis results from bronchial obstruction, lung sliding is typically absent. However, in compression atelectasis, as that induced by pleural effusion, lung sliding may still be present, particularly in the early phase, before the air is completely reabsorbed [68].

### Pneumonia

**Fig. 4:**
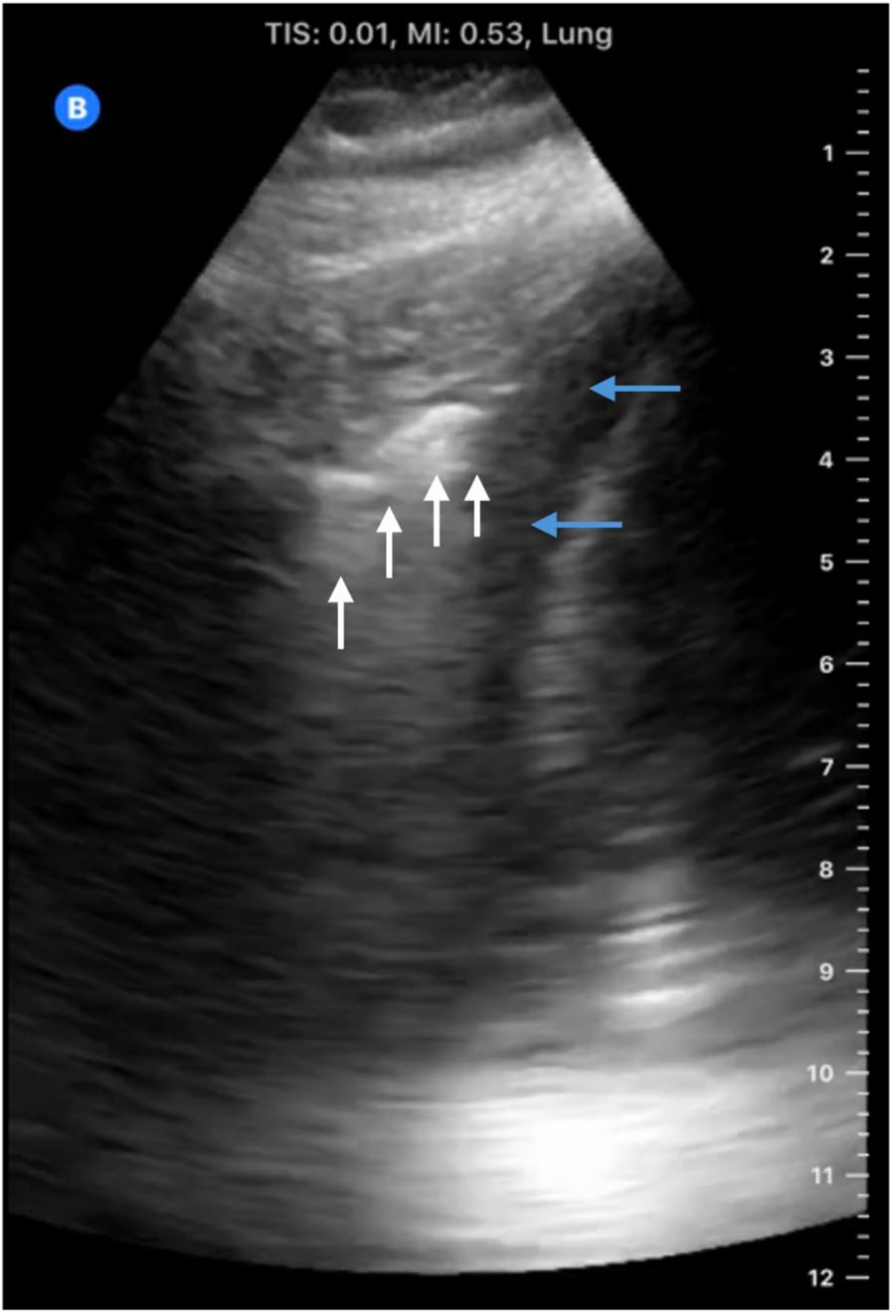
Consolidated lung tissue. The irregular border “Shred sign” between the consolidated and normal lung tissue (white arrows). The lung here is surrounded by an pleural effusion (blue lines)

LUS is an excellent tool for diagnosis of pneumonia having a sensitivity of about 90% and specificity of 98% [44, 69]. The loss of lung aeration in pneumonia allows the ultrasound beam to be transmitted beyond the pleural line, and the consolidated lung appears as hypoechoic tissue that is wedge-shaped and is usually poorly defined [40]. This results in a tissue-like density described as “pulmonary hepatization” [25, 33, 41, 44, 70]. Unilateral B-lines, thickening, and irregularities of the pleural line may also be seen; however, it should be noted that if the consolidation does not reach the pleura, it will not be visualized with LUS causing a false negative result for pneumonia [70–72]. An irregular border often exists between the consolidated and normal lung tissue and is sometimes appreciated by ultrasound resulting in what is known as the “shred sign” (Fig. 4) [7].

Air bronchograms can be characterized as static or dynamic, which refers to whether the hyperechoic circles are stationary or move with respiration, respectively, with the latter indicating an ongoing airflow that is more likely associated with consolidation rather than atelectasis [23, 40, 41, 66, 67, 70, 73, 74]. In the case of static air bronchograms, the presence of pulmonary blood flow on color Doppler imaging can help to differentiate pneumonia from atelectasis as blood flow is more likely to be absent in atelectasis compared to pneumonia [67]. This is because in atelectasis, the collapsed normal lung is likely to exhibit hypoxic pulmonary vasoconstriction that leads to reduced intrapulmonary shunt or blood flow that may be challenging to observe or absent on color Doppler; whereas, in pneumonia, intrapulmonary shunting or blood flow is more likely to be visualized on color doppler because the inflammation impairs hypoxic pulmonary vasoconstriction [75–77]. It is important to note that the overall diagnostic accuracy of LUS utilizing color Doppler to differentiate pneumonia and atelectasis is significantly improved when considering the clinical presentation as previously shown when combined with the simplified Clinical Pulmonary Infection Score (sCPIS) (composed of temperature, leukocyte count, tracheal secretions, PaO2/FiO2, and chest radiography), particularly in the setting of static air bronchograms (typically associated with atelectasis) and the presence of intrapulmonary blood flow observed on color doppler where pneumonia was more likely than atelectasis when a combined LUS-sCPIS score (composed of temperature, leukocyte count, tracheal secretions, PaO2/FiO2, and color Doppler intrapulmonary shunt) was elevated [67, 78].

LUS can also help in distinguishing between viral and bacterial pneumonia with high accuracy [79–81]. In viral pneumonia, the areas of consolidation are typically subpleural in location, smaller (< 0.5 cm), multiple, bilateral and associated with pathologic B-lines, defined as three or more B-lines in one interspace or the presence of a confluent B line that occupies most of the interspace [82]. Conversely, in bacterial pneumonia, consolidation tends to be unilateral, larger in size and associated with air bronchograms; these findings may be present in patients with viral pneuomonia, ARDS, and other respiratory pathologies with superimposed bacterial pneumonias. It is also important to highlight that scanning the dorsal zones of the lungs is crucial when assessing for bacterial pneumonia in bedridden supine patients as the disease often affects the dorsal lung regions [70, 71]. During the COVID-19 pandemic, LUS has become increasingly recognized for its significant role in diagnosing viral pneumonia. The availability, practicality and the ease of sanitization of ultrasound have facilitated not only the diagnosis of COVID pneumonia but also allowed the grading of clinical severity, prediction of disease progression and the need for invasive ventilation [5, 83–91].

### Pulmonary edema

LUS is superior to conventional chest radiography for the detection of pulmonary edema [11, 92–94]. The hallmark LUS finding with pulmonary edema is the presence of [82] B-lines (Supplemental Fig. 9) defined as three or more B-lines in one interspace or the presence of a confluent B-line that occupies most of the interspace. The number of B-lines present is correlated with the degree of loss of lung aeration and the presence of interstitial and alveolar edema characterized by ground glass opacities on computed tomography (CT) [40, 49, 95].

With pulmonary edema, lung sliding is preserved, and the finding of pathologic B-lines is usually bilateral and homogenous. This helps differentiate pulmonary edema from other lung pathologies associated with pathologic B-lines as in pneumonia, acute respiratory distress syndrome and lung contusion [49, 96, 97]. Another key distinguishing feature is pleural line abnormalities which are absent in pulmonary edema of cardiac etiology and present in other noncardiogenic lung pathologies (such as interstitial lung disease, idiopathic pulmonary fibrosis, nonspecific interstitial pneumonia, and acute interstitial pneumonia) associated with pathologic B-lines which also exhibit a fragmented pleural line and vertical subpleural pattern on M-mode [98, 99].

A potential theoretical application of LUS is in fluid resuscitation of patients in shock, where the appearance of B-lines may signify the development of pulmonary edema. This assessment may be helpful in characterizing the benefit-to-risk ratio of additional fluids administration, as proposed in the FALLS protocol by Lichenstein et al [25]. However, it is crucial to note that there is no rigorous data directly linking the occurrence of B-lines with worsening respiratory status following fluid administration. It is important to note the limitations of using pathologic bilateral B-lines to guide fluid resuscitation as they are non-specific for pulmonary edema and can also be seen in viral pneumonia (such as COVID-19), ARDS, and interstitial lung disease; and bilateral B-lines present more often with bilateral pleural effusions in the setting of cardiogenic pulmonary edema [100, 101]. While B-lines may suggest an increase in interstitial fluid, it is critical to interpret these findings in the patients’ broader clinical context and that B-lines should be evaluated alongside other clinical indicators of volume status and cardiac function to effectively guide fluid management decisions. In patients presenting with pathologic bilateral B-lines that may not be secondary to cardiogenic pulmonary edema, dynamic indices of volume responsiveness may be helpful for guiding fluid management while weighing the risks and benefits of fluid administration in the patients’ broader clinical context [102, 103]. It is important to note that even in cases of cardiogenic pulmonary edema, hypotensive patients may be hypovolemic and benefit from fluid administration as previously described by Figueras and Weil et al [104]. For example, patients experiencing cardiogenic shock may require fluids, as acute pulmonary edema such as in the case of flash pulmonary edema from impaired left ventricular diastolic dysfunction can lead to a reduction in the effective circulating blood volume [105]. Echocardiography and hemodynamic monitoring are valuable tools for assessing systolic and diastolic function, as well as dynamic measures of volume responsiveness, to optimize cardiac performance and guide therapies such as inotropes, vasodilator, vasopressor, fluid, and diuretic use to improve patient care while preventing further pulmonary edema within the patients' broader clinical context [106–108].

LUS is also important in differentiating between congestive heart failure and COPD exacerbation. In the former, there will be bilateral homogenous pathologic B-lines on LUS, often associated with the presence of pleural effusion, while with the latter, the predominant finding will be multiple A-lines [7, 25, 44, 109–111]. This distinction will be described in more detail in the “examination protocol” section of this article. Another important application is the diagnosis of pulmonary edema during spontaneous breathing trial, where the appearance or the increase in number of B-lines may signify an increased risk of weaning failure [112].

### Acute respiratory distress syndrome (ARDS)

In ARDS, the ultrasound findings depend on the severity and stage of the disease. Early in the exudative phase of the disease, there is an accumulation of alveolar and interstitial fluid with findings of pathologic B-lines characteristic of pulmonary edema but with a more heterogeneous distribution [113–115]. These LUS findings are followed either by recovery or the development of poorly defined hypoechoic areas similar in appearance to consolidation with dynamic air bronchograms and with occasional loss of lung sliding and the presence of an irregular pleural line in the affected areas [49, 99, 100, 113, 116–120].

The importance of LUS has been highlighted in the new global definition of ARDS where LUS by a well-trained operator has been added as one of the imaging modalities to diagnose bilateral opacities in ARDS [121]. LUS is not only important in diagnosis, but it can also be used to predict and monitor response to various interventions as lung recruitment and prone positioning [122]. For example, the presence of areas of normally aerated lung and lower LUS score (described below) were found to predict a more favorable response to prone positioning [123–125].

### Pleural effusion

LUS imaging of a pleural effusion will result in an anechoic space in the dependent lung regions between the parietal and visceral pleura (Supplemental Fig. 10) [7, 41, 49]. LUS has a sensitivity of 83–100% and specificity of 93–100% in the diagnosis of pleural effusion [7, 126, 127].

In the absence of any pleural fluid, extension of the thoracic spine above the diaphragm should not be visualized with LUS as air prevents the transmission of the ultrasound beam; however, in the presence of an effusion, the pleural fluid will create an acoustic window through which the spine can be seen (Supplemental Fig. 10). This LUS finding is referred to as the “spine sign” and has a sensitivity and specificity of 92.9% and 87.5% respectively for the presence of a pleural effusion [128, 129]. Also, in the absence of pleural fluid, the expansion of the lung during inspiration pushes the diaphragm down and this has been described as “curtain sign”. This sign is absent in pleural effusion and has been described as the “absent curtain sign” [28].

LUS can also be helpful in determining the nature of the pleural fluid and differentiating between a transudative and an exudative effusion. Pleural effusions that are complex-appearing, septated or with echogenic patterns are almost always exudative in nature. The “plankton sign” which describes floating debris in complex effusions that appear as punctiform opacities that move with respiration and cardiac pulsations and is usually associated with exudative effusions [24]. In patients with malignancies, these echogenic floating particles that move with respiration and cardiac pulsations has been associated with malignant pleural effusions [130]. It is important to note that a simple anechoic appearance of an effusion has a low predictive value of identifying a transudative effusion [131–135].

Visualization of lung parenchyma floating within the pleural fluid is referred to as the “sinusoidal sign” (Supplemental Fig. 10). The sinusoidal sign is commonly seen in effusions but can be absent if the volume of effusion is large enough to displace the lung from the ultrasound view [7, 41]. The sinusoid sign may also be absent in trapped lung pathology such as in the case of empyema and malignant effusions where the formation of fibrous pleural peels prevents re-expansion of the lung. Supplemental Video 3 highlights examples of different LUS signs in pleural effusion [28]. Estimation of pleural fluid volume can be done through the application of different published formulas; however, a more pragmatic approach involves categorizing pleural effusion into small, moderate or large. In general, an effusion depth of more than 4–5 cm typically corresponds to a volume exceeding 1000 ml and more likely to be clinically relevant [133, 136].

### Contusion

Signs of lung contusion on LUS include an increase in the number of B-lines in the affected lung regions as well as the presence of consolidation. Ultrasound can diagnose lung contusion earlier than a conventional chest x-ray with a much higher sensitivity and specificity [49, 137–139].

## Examination protocols, scoring systems and a framework for LUS

Several protocols have been developed to facilitate a structured approach to LUS. Most of these protocols also incorporate a more comprehensive ultrasound examinations of the heart, abdomen and venous system to aid in patient diagnosis and timely management. A full discussion of these applications is beyond the scope of this article but have been recently reviewed [7, 11, 25, 44, 140].

One of the most commonly used LUS protocols is the “the bedside lung ultrasound in emergency” (BLUE) protocol which is used for the rapid diagnosis of acute respiratory failure and includes lung as well as vascular ultrasound when indicated. It is integrated with vital signs and other clinical data for maximal efficiency [7, 25, 44, 140, 141]. The BLUE protocol provides accuracy approaching 90% (81%-100%) in diagnosing different lung pathologies such as pulmonary edema, pulmonary embolism, pneumonia, pneumothorax, and COPD or asthma, however it is worth mentioning that it was a single-center study by two highly experienced operators [25].

**Fig. 5:**
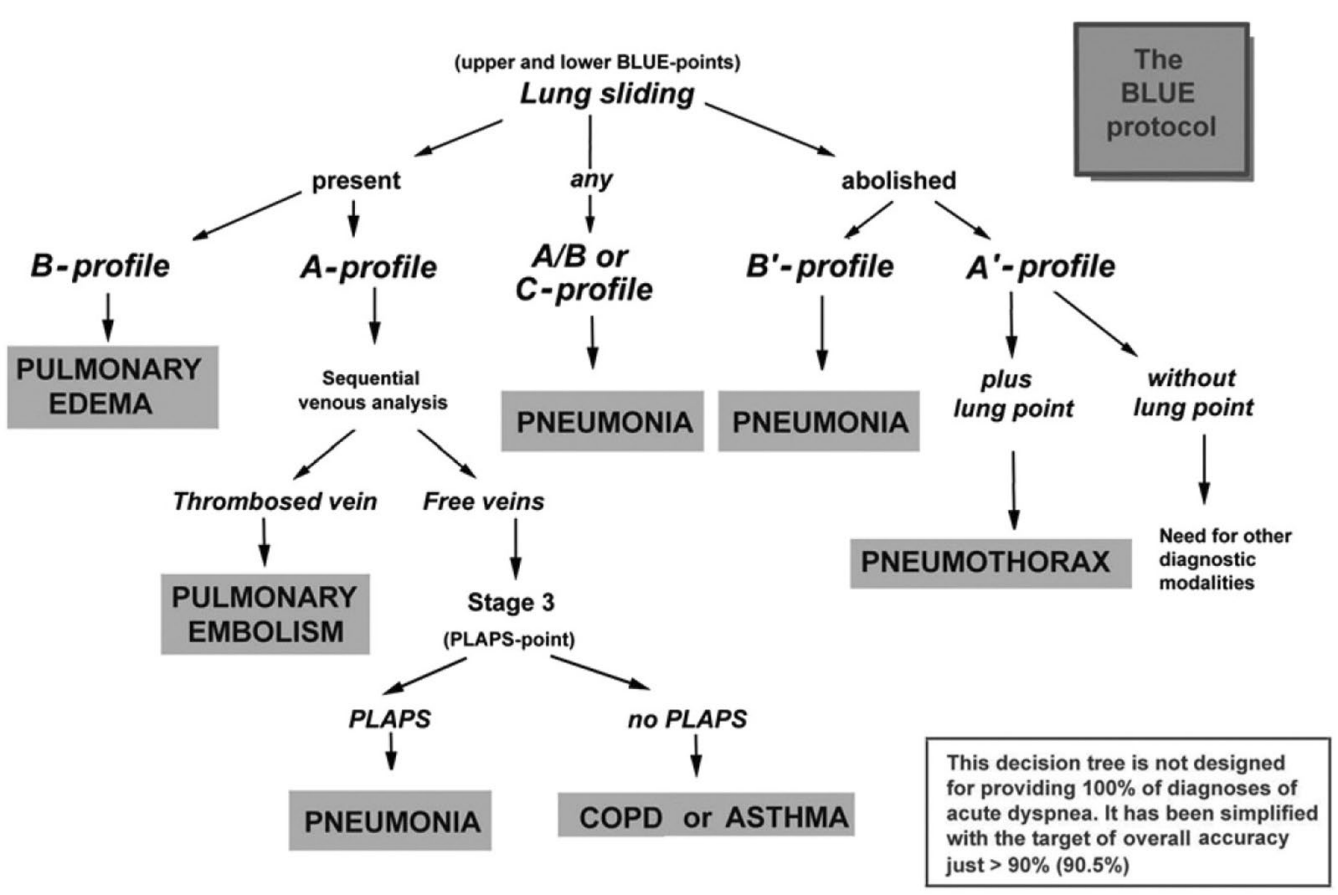
The Bedside Lung Ultrasound in Emergency (BLUE) Protocol for patients with acute respiratory failure. **A-profile**: Lung sliding + A-lines, **B-profile**: Lung sliding + B-lines, **A/B-profile**: A-profile in one lung and B-profile in the other, **C-profile**: Anterior lung consolidation, **A’-profile**: A profile + negative lung sliding, **B’-profile**: B profile + negative lung sliding. Reproduced with permission from Dr. Lichtenstein [7, 25]

The (BLUE) protocol, first published in 2008, consisted of scanning 3 zones on each side (anterior, lateral and posterolateral chest walls) and each zone was further divided into upper and lower halves resulting in 6 scanning points on each side[44]. This protocol was then modified into a more simplified approach consisting of scanning at only three standardized points called the “Blue points” seen and described in Supplemental Fig. 11. The initial step in the modified BLUE-protocol is looking for lung sliding on both sides. The algorithm in Fig. 5 is then followed, and this categorizes the patients into different disease profiles including pulmonary edema, pulmonary embolism, pneumonia, pneumothorax, and COPD or asthma [7]. An *A-profile* associates Lung sliding with A-lines, a *B-profile* associates Lung sliding with B-lines, an *A/B-profile* is characterized by an A-profile in one lung and B-profile in the other, a *C-profile* is characterized by the presence of Anterior lung consolidation, an *A’-profile* is an A profile with negative lung sliding, and finally a *B’-profile* which is B profile with negative lung sliding [7, 25]. It is important to note that for many practical algorithms that facilitate clinical diagnoses, including the BLUE protocol, there is often significant overlap between findings seen in various pathological conditions making dichotomic categorization challenging. For instance, it can be challenging to difference between cardiogenic pulmonary edema and ARDS given that the B-profile may be seen in early ARDS before it progresses towards a consolidative pattern (C-pattern) as it progresses towards severe ARDS [100, 142, 143].

Another LUS protocol is the “Fluid administration limited by lung sonography” (FALLS) protocol. This protocol was initially developed to guide fluid resuscitation in unstable patients through the use of real-time cardiac and LUS to sequentially assess for obstructive, cardiogenic, hypovolemic, and distributive shock. This protocol uses the emergence of pathologic B-lines on LUS as the end point of fluid resuscitation, however, in one study an increase in the number of B-lines has been shown to have a sensitivity of 80% and specificity of only 57% in distinguishing between patients who responded to fluid challenge and those who didn’t respond (Supplemental Fig. 12) [25, 144]. It is important to note that there are significant limitations with using the emergence of pathologic B-lines on LUS to guide fluid resuscitation given that evolving bilateral B-lines are non-specific for pulmonary edema and may also be seen in other pathologies such as viral pneumonia, ARDS, pulmonary contusions, and acute interstitial lung disease.

Different scoring systems have also been developed to grade the severity of lung pathologies. This allows healthcare providers to use LUS quantitively, not only for diagnosis, but also to monitor disease progression and the response to therapy [145–148]. The discussion of different scoring systems and their application in clinical practice are beyond the scope of this article, but one of the most frequently used ones is the “lung ultrasound score”. In this scoring system, each lung region examined is given a score from 0 to 3 depending on the degree of loss of aeration where a score of 0 represents normally aerated lung with no more than two B-lines and a score of 3 represents complete consolidation of the lung parenchyma. The scores of lung regions examined are then added and is used for follow-up of disease progression and response to therapy such as antibiotic treatment, lung recruitment and prone positioning [123, 124, 145, 149].

One of the LUS frameworks is the I-AIM framework (Indication, Acquisition, Interpretation and Medical decision-making) [28, 150]. It follows a stepwise approach for performing a focused sonographic examination. A systematic approach to LUS using the I-AIM framework has been described by Kruisselbrink et al. and represent a practical way of using LUS to diagnose different pathologies [28]. It starts with having an indication for LUS (respiratory symptoms of unknown etiology or unexplained radiographic findings), followed by LUS image acquisition (patient positioning, probe selection, picture analysis and an examination protocol), then interpretation (presence or absence of suspected findings, generating a differential diagnosis and focused examination of other structures if needed) and finally ending with a medical decision making in light of the patient’s history, physical exam findings, lab and radiological data [28]. It is also essential to store the acquired images and report LUS findings for follow-up and medicolegal purposes. Supplemental Fig. 13 shows a sample LUS report from Kruisselbrink et al. [28].

## Training and achieving competence in lung ultrasound

The use of LUS is rapidly growing, and its growth was accelerated by the COVID-19 pandemic [5, 151]. Its diagnostic value is not restricted to physicians but also includes other healthcare professionals as advanced practice providers, paramedics and respiratory therapists [14, 15, 152–154]. Widespread application of LUS to the daily practice will add additional tools to these providers’ armamentarium to help change the medical care strategy that may translate to improved patient care outcomes [155]. However, to ensure the proper use of LUS, structured training programs are of paramount importance because if adequate training is not provided, serious diagnostic errors could result which may adversely impact clinical decision making. Supplemental Table 1 discusses some of these major societal programs’ requirements and recommendation specific to LUS [156–165]. The supplemental text includes detailed information related to training and achieving competence in lung ultrasound.

## Conclusion

LUS is becoming an essential tool for bedside diagnosis of a variety of lung pathologies. Characteristic findings are well characterized and can be easily learned by critical care, pulmonary, and anesthesia providers, as well as respiratory therapists and other health care providers. A variety of LUS protocols exist and provide a structured approach to diagnosis. It is a non-invasive and a very reliable tool that can be used by members of the patient’s treatment team to more quickly and accurately diagnose and treat the underlying etiology of their patient’s respiratory issues.

## Supplementary Information

Normal LUS signs. Reproduced from Kruisselbrink et al. with permission from Dr. Goffi28.

LUS signs in Pneumothorax. Reproduced from Kruisselbrink et al. with permission from Dr. Goffi28.

Common LUS signs in Pleural effusion. Reproduced from Kruisselbrink et al. with permission from Dr. Goffi28.

Additional file 4

Additional file 5

Additional file 6

## Declarations

### Data availability

No datasets were generated or analysed during the current study.
