# Supplementary material for: Nuts and bolts of lung ultrasound: utility, scanning techniques, protocols, and findings in common pathologies
Source: Crit Care. 2024 Oct 7;28:328. doi: 10.1186/s13054-024-05102-y (PMC11460009; doi:10.1186/s13054-024-05102-y)
Supplement: Supplementary file 4 — Additional file 4 [file 13054_2024_5102_MOESM4_ESM.docx]

**Training and achieving competence in Lung Ultrasound**

Up to this time, none of the major scientific medical societies offer a dedicated LUS training course nor is there a separate LUS certification. Most of LUS training programs available are done as a part of POCUS training which includes cardiac, gastric, vascular and abdominal ultrasound as well. The curricula for these training programs include a combination of online e-learning modules, in-person ultrasound didactics and hands-on training, completion of online portfolio, preceptorship, online image interpretation training, practical scanning assessment on real-life model or simulator, oral assessment of case studies, case logbook, and/or written and practical examinations. Supplemental Table 1 discusses some of these major societal programs’ requirements and recommendations specific to LUS^146-156^.

In addition to training on patients and volunteers, simulation has also been used as it provides opportunities for structured training and assessment without having to expose anyone to any discomfort but can also be expensive and unrealistic depending on the degree of fidelity of the simulator^157-159^. It is important to note that Pietersen et al. found in their randomized controlled trial of simulation-based training versus training on health volunteers that simulation-based training in thoracic ultrasound did not improve clinical performance compared to conventional hands-on training on health volunteers, but simulation-based training was significantly better than the control group without hands-on training^159^. Another tool for teaching LUS is animal lab training where different respiratory pathologies can be induced in animal models and trainees are trained to identify different LUS signs^160,161^. Human cadaver models have also been used for training in LUS ^162^. There is also a wealth of information available for LUS online like the Perioperative Interactive Education (PIE) website designed by the Anesthesia department in Toronto General Hospital which offer an interactive application for LUS^163^.

The number of LUS specific exams, if specified at all, varies between different training programs. The European Society of Intensive Care Medicine’s European Diploma in Advanced Critical Care in Echocardiography and National Board of Echocardiography’s Advanced Critical Care Echocardiography Certification Pathway require a minimum number of Cardiac ultrasounds (130-150) but do not stipulate a minimum number of lung ultrasound studies. The American Society of Anesthesiology’s Diagnostic POCUS Certificate Program requires at least 30 lung ultrasound images without any specification for the required lung pathologies. The Society of Critical Care Medicine’s Critical Care Ultrasound (CCUS) with Focused Ultrasound Pathway includes five procedural and twenty diagnostic LUS exams. The British Society of Echocardiography/Intensive Care Society’s Adult Critical Care Echocardiography Accreditation requires at least 10 cases of refractory hypoxemia and difficult weaning. The American College of Chest Physicians’ (CHEST) Point of Care Ultrasound Certificate of Completion requires completion of an online portfolio consisting of five studies with normal A-lines, consolidation, pleural effusion, and B-lines, and their Critical Care Ultrasonography Certificate of Completion requires four images of pleural effusion, lung sliding and consolidation. Both the Society of Point of Care Ultrasound’s POCUS Practice Guidelines for Competency and The American College of Emergency Physician’s Residency or Practice Based Pathway for Emergency Ultrasound recommends completion of 25-50 exams in each domain (i.e., LUS).

The requirements from many of these major societies (Supplemental Table 1) are in line with findings from prior studies that have shown that the number of exams performed by physician and non-physicians to achieve competency in LUS ranges between 5-30 exams^164-167^. Of note, there have been a couple studies that have specifically targeted respiratory therapists for LUS training. In one study, it was found that RTs without any previous experience can perform competently and independently perform LUS after at least ten directly supervised scans^168^. In another study, RTs achieved competence in acquisition and interpretation of upper lung zone images from 10 initial scans but more experience was required for interpretation of lower lung zone images^169^.

Competency is assessed by reviewing one’s portfolio by an expert in ultrasound and many major societies also require passing a summative assessment as well (Supplemental Table 1). The summative assessment is typically a written exam. Some societies such as the British Society of Echocardiography/Intensive Care Society’s Adult Critical Care Echocardiography Accreditation, European Society of Intensive Care Medicine’s European Diploma in Advanced Critical Care Echocardiography, and European Respiratory Society also require a practical examination on a real-life model or simulator as well. The British Society of Echocardiography/Intensive Care Society’s Adult Critical Care Echocardiography Accreditation is unique in that it also requires a viva (ie. oral) assessment of five separate patient case studies. The American College of Emergency Physicians’ Residency or Practice Based Pathway for Emergency Ultrasound, The Society of Point of Care Ultrasound’s POCUS Practice Guidelines for Competency, and the Society of Critical Care Medicine’s Critical Care Ultrasound with Focused Ultrasound Pathway do not mandate a summative exam.

Many major societies promote the need for ultrasound certification to maintain patient safety and minimize misdiagnosis. There is little evidence to support that extramural certification improves patient care and safety^170^. In fact, some societies such as The Society of Point of Care Ultrasound argue that while it is important for providers to demonstrate competency, extramural certification and testing is not necessary given the significant amount of costs associated with sitting for an exam, the non-clinical time needed to prepare for the exam, time spent away from clinical activities to attend costly ultrasound training course. All of these factors can often times serve as a significant barrier to performing and practicing ultrasound.

Aside from the costs associated with certification noted above, a lot of barriers still exist for the widespread training and application of LUS. Lack of dedicated clinical time to devote ultrasound towards training and lack of trained supervisors are among the most common^171^. The use of online learning modules and online portfolios that can be reviewed by a remote expert has helped to relieve many of these barriers. There are also no specific competency requirements, accreditation programs or certifying exams specifically for LUS. It is important to note that Pieteresen et al. and Skaarup et al. have published expert panel-developed and validated assessment tools for clinical competency in lung ultrasound^172,173^.

In the future, more work and research should be directed to overcome these barriers. Dedicated LUS training courses, competency requirements, and certifying exams need to be developed. These courses should not target only physicians but also other healthcare providers involved in patients’ care and should be easily attainable at an affordable cost. This will allow the widespread application of LUS in our daily practice.
