# Supplementary material for: Nuts and bolts of lung ultrasound: utility, scanning techniques, protocols, and findings in common pathologies
Source: Crit Care. 2024 Oct 7;28:328. doi: 10.1186/s13054-024-05102-y (PMC11460009; doi:10.1186/s13054-024-05102-y)
Supplement: Supplementary file 5 — Additional file 5 [file 13054_2024_5102_MOESM5_ESM.docx]

**Supplemental Figure Legends**

**
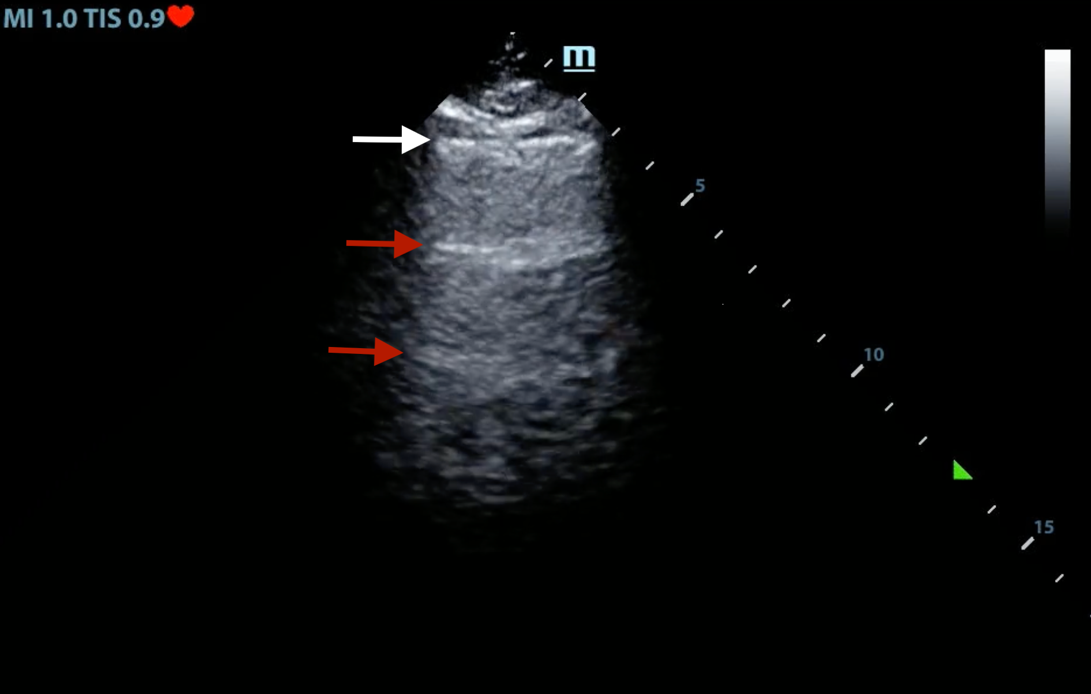
**

**SUPPLEMENTAL FIGURE 1.** Pleural line (White arrow) and A-lines (red arrows) appear as a reverberation artifact from the pleural line.

**
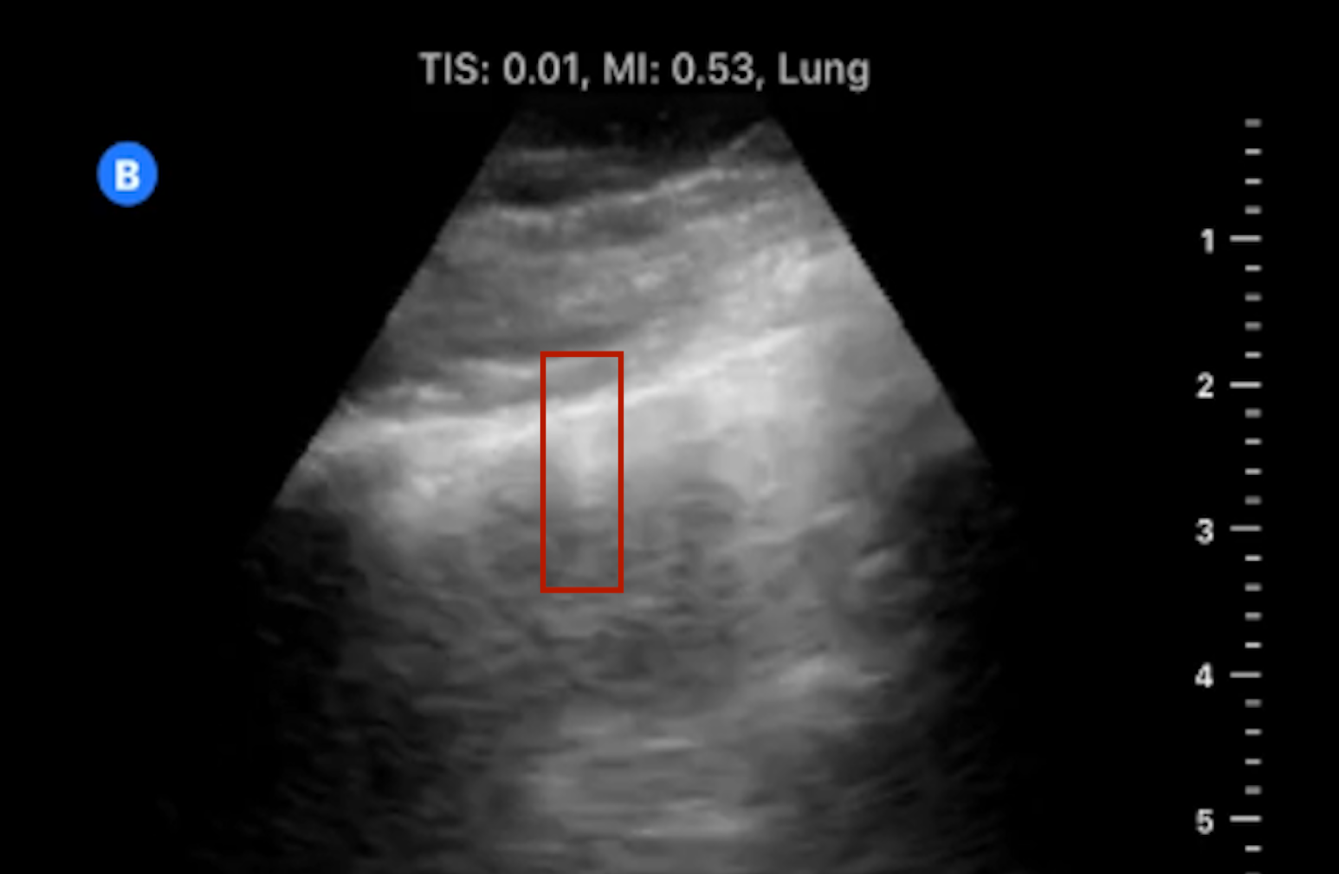
**

**SUPPLEMENTAL FIGURE 2.** Short vertical artifact extending for a short distance beyond the pleural line and not considered a B-line.

**
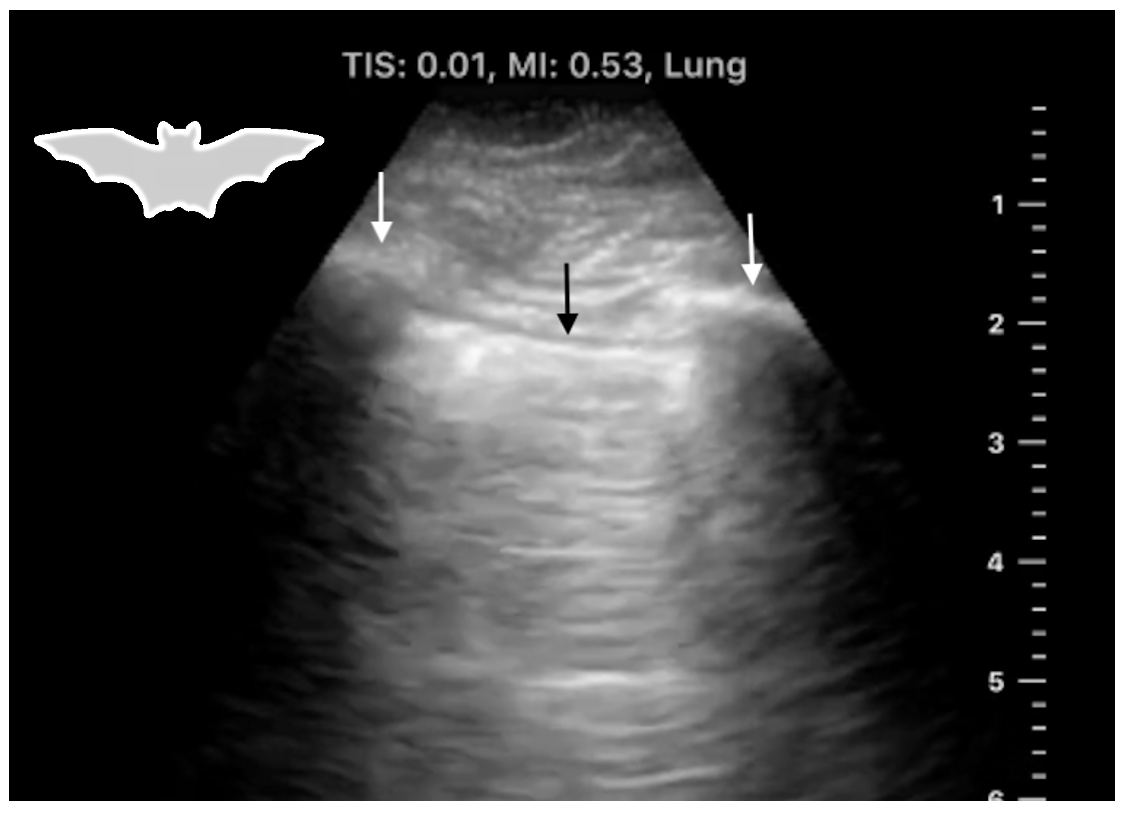
**

**SUPPLEMENTAL FIGURE 3.** The Bat Sign: The ribs (white arrows) and the Pleural line (black arrow) outlining the silhouette of a bat.

**
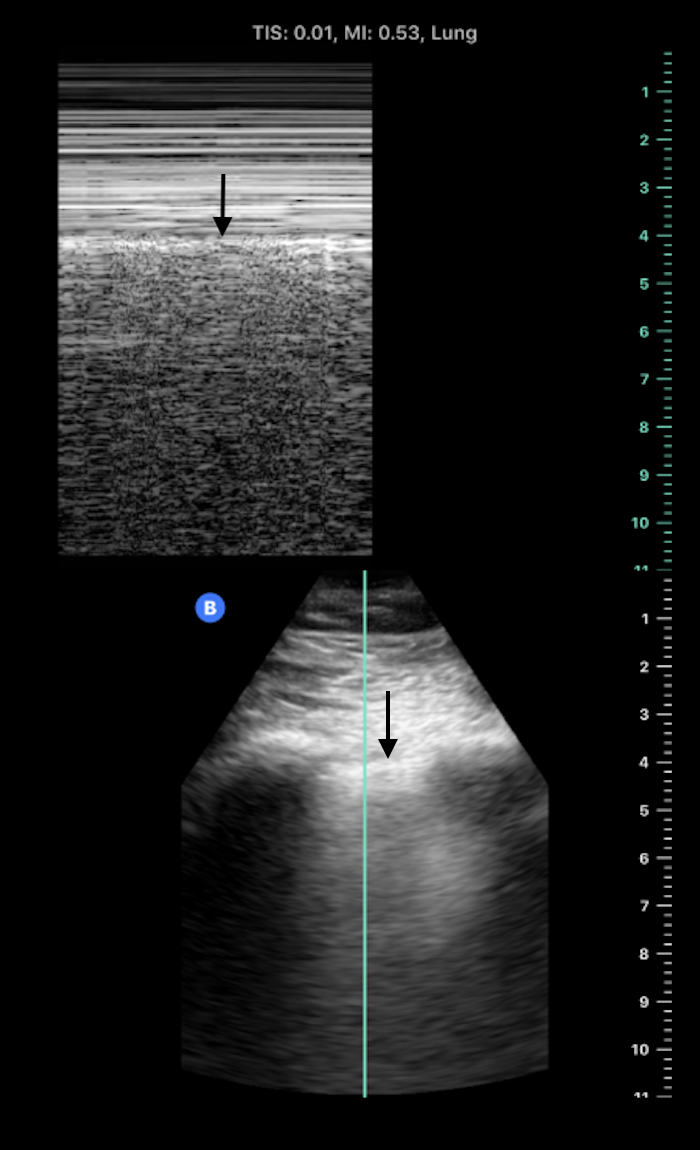
**

**SUPPLEMENTAL FIGURE 4.** Top panel: M-mode with Pleural line (black arrow) and obvious different patterns on both sides. Superficial to the pleural line are motionless horizontal lines and deep to the pleural line is a “sandy beach” pattern generated by pleural sliding creating a “seashore sign”. Bottom panel: The 2D image of normal lung sliding with the pleural line denoted by the black arrow.

**
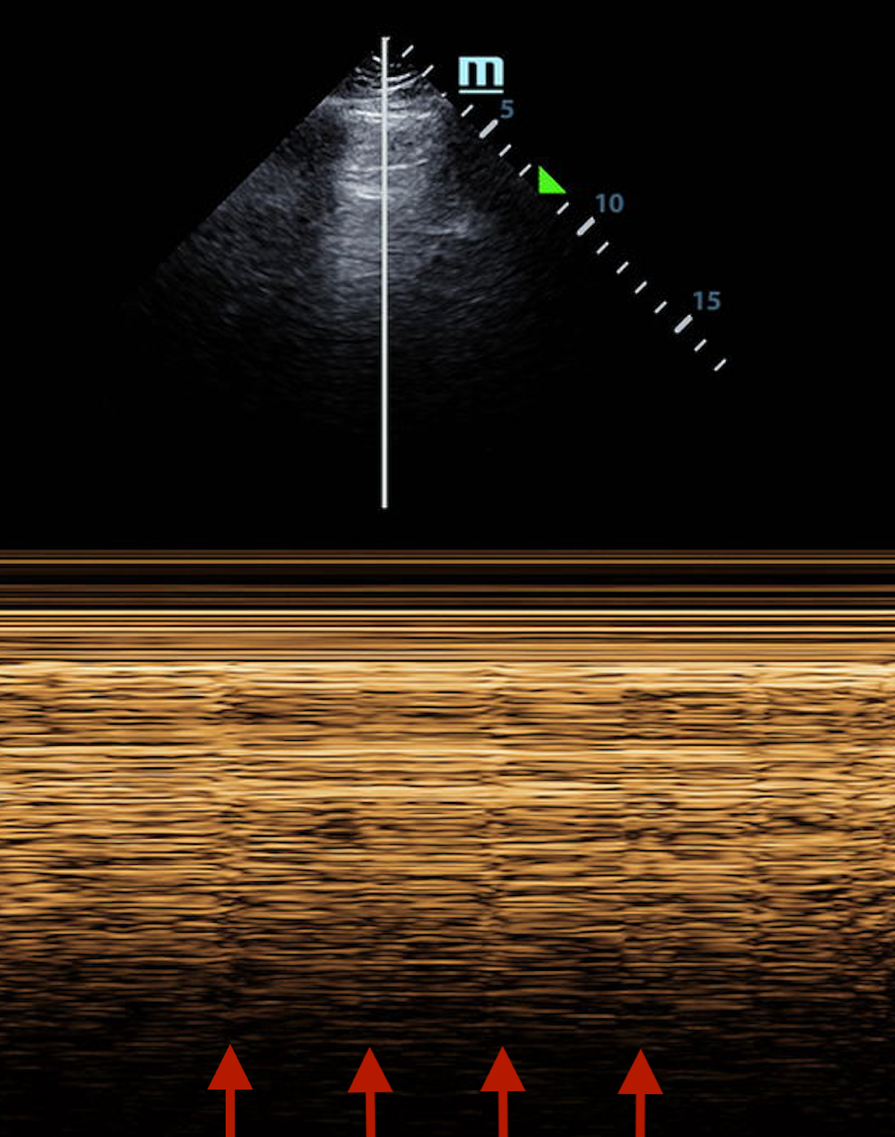
**

**SUPPLEMENTAL FIGURE 5.** Lung Pulse visualized in M-mode (vertical red arrows). The presence of such sign rules out pneumothorax.

**
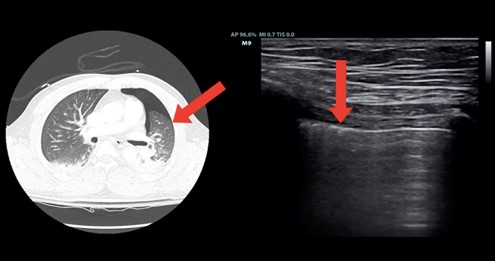
**

**SUPPLEMENTAL FIGURE 6.** Lung point seen on CT and 2D image of LUS (red arrows). As previously mentioned, the presence of “lung pulse” and B-lines will rule out pneumothorax with a very high sensitivity as air between the visceral and parietal pleura will prevent the transmission of cardiac oscillations and detection of alveolar and interstitial fluid. Image reproduced with permission from Dr. Goffi ^28^.

**
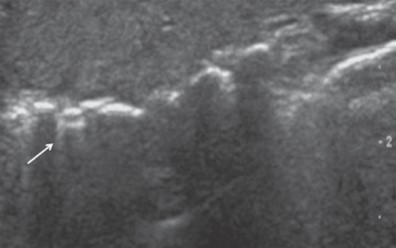
**

**SUPPLEMENTAL FIGURE 7.** E-lines (white arrow) arising from the air in subcutaneous tissue, not from the pleural line. Figure reproduced from Francisco et al^63^.

**
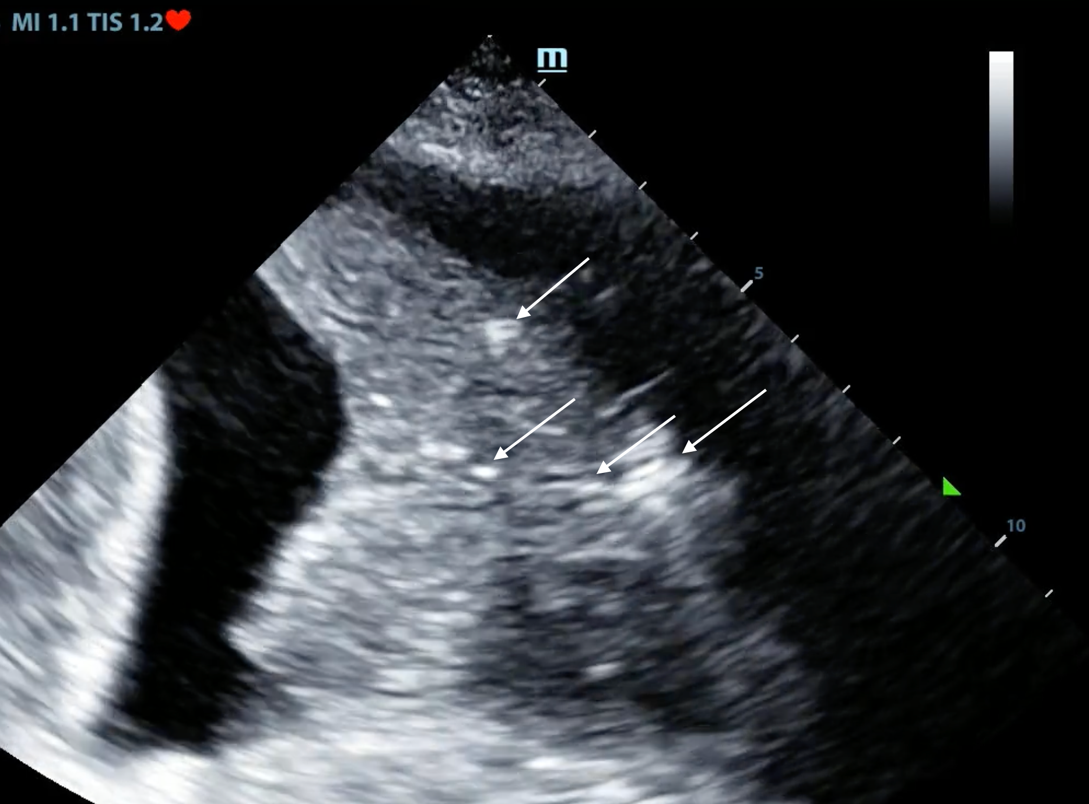
**

**SUPPLEMENTAL FIGURE 8.** Lung ultrasound showing “air bronchogram” (white arrows) and anechoic pleural effusion.

**
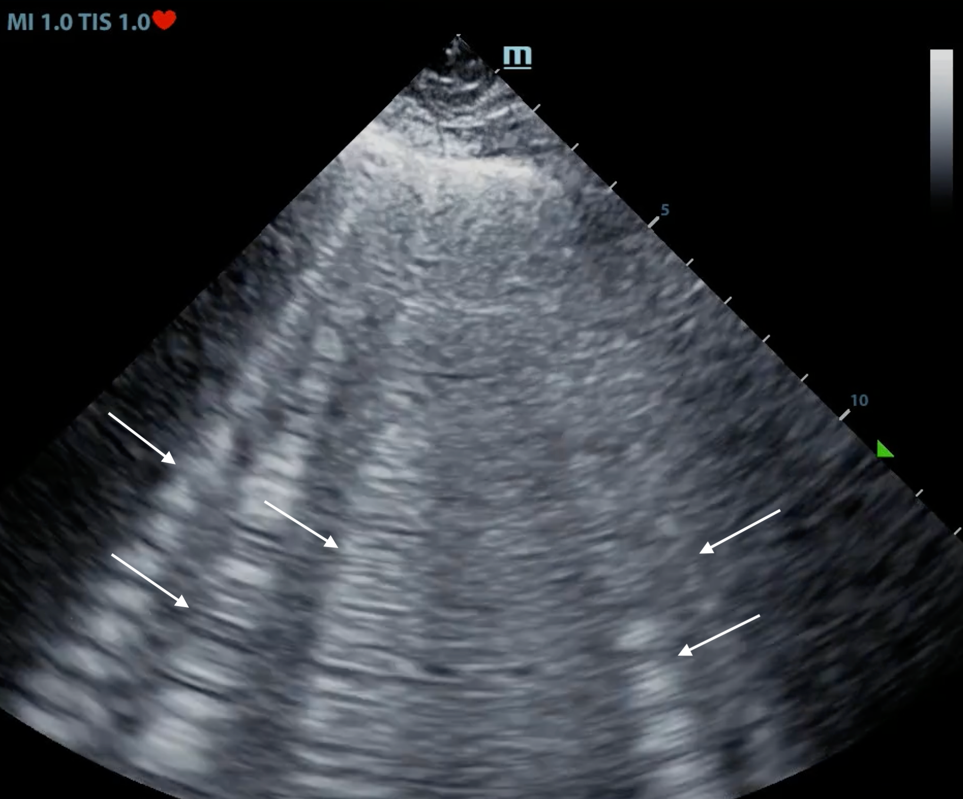
**

**SUPPLEMENTAL FIGURE 9.** Pathologic B-lines in Pulmonary edema. Note that the B-lines extend from the pleural line all the way down to the bottom of the screen. Three or more B-lines are considered pathological^82^.

**
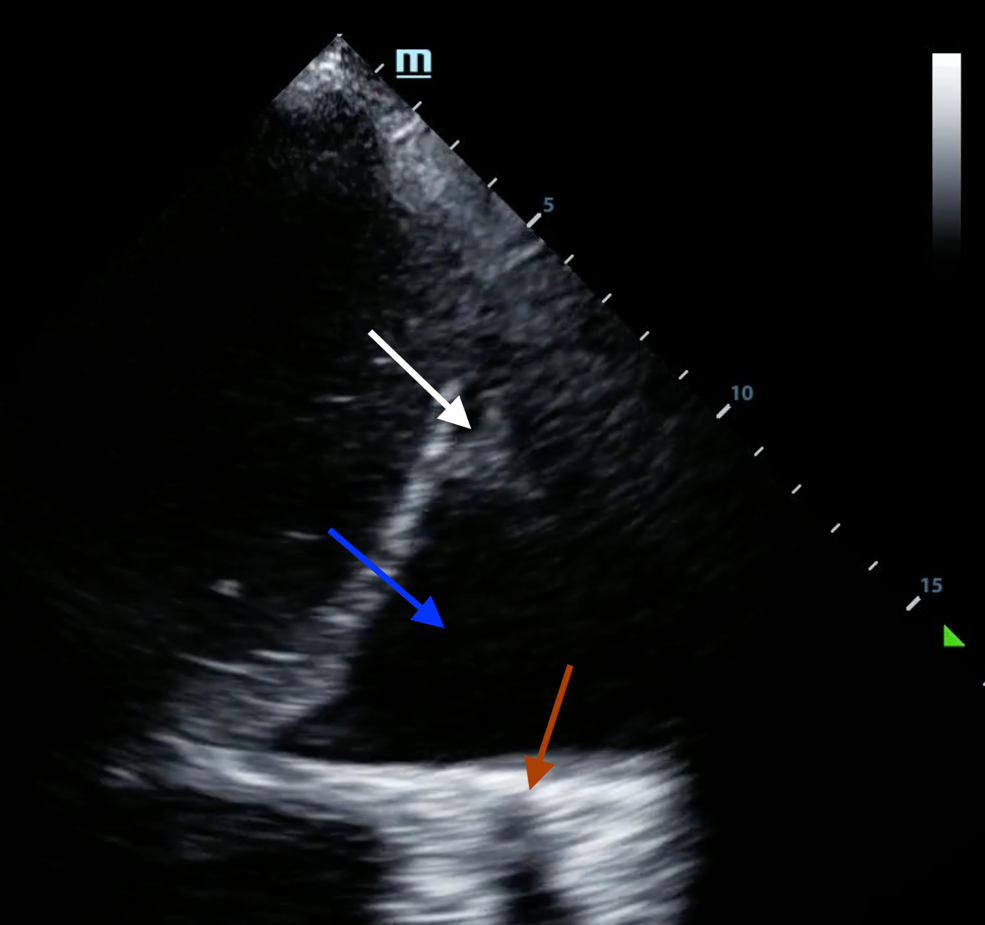
**

**SUPPLEMENTAL FIGURE 10.** Pleural Effusion (blue arrow) with underlying collapsed lung (white arrow). Also note that the thoracic spine is visualized with LUS “Spine sign” (red arrow).

**
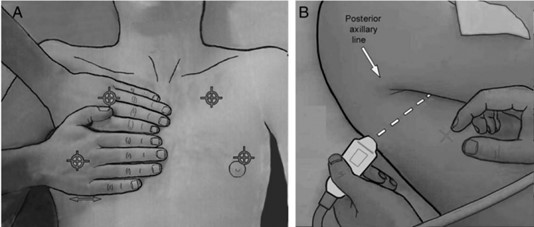
**

**SUPPLEMENTAL FIGURE 11.** The BLUE points. The location of the upper and lower blue points is determined by placing 2 hands, roughly of the same size as the patient’s hands, with the little finger of the upper hand just under the clavicle, fingertips at the midline and the lower hand below the upper hand with the thumbs of both hands excluded. The upper blue point will be in the middle of the upper hand and the lower blue point will be in the middle of the palm of the lower hand. The third blue point is called the Posterolateral alveolar and/or pleural syndrome point (PLAPS point). This point is located at the intersection of a horizontal line at the level of the lower blue point and a vertical line at the posterior axillary line. Reproduced with permission from Dr. Lichtenstein^7,25^.

**
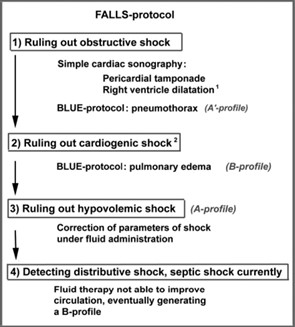
**

**SUPPLEMENTAL FIGURE 12.** Fluid Administration Limited by Lung Sonography (FALLS) Protocol. Reproduced with permission from Dr. Lichtenstein^25^.

**
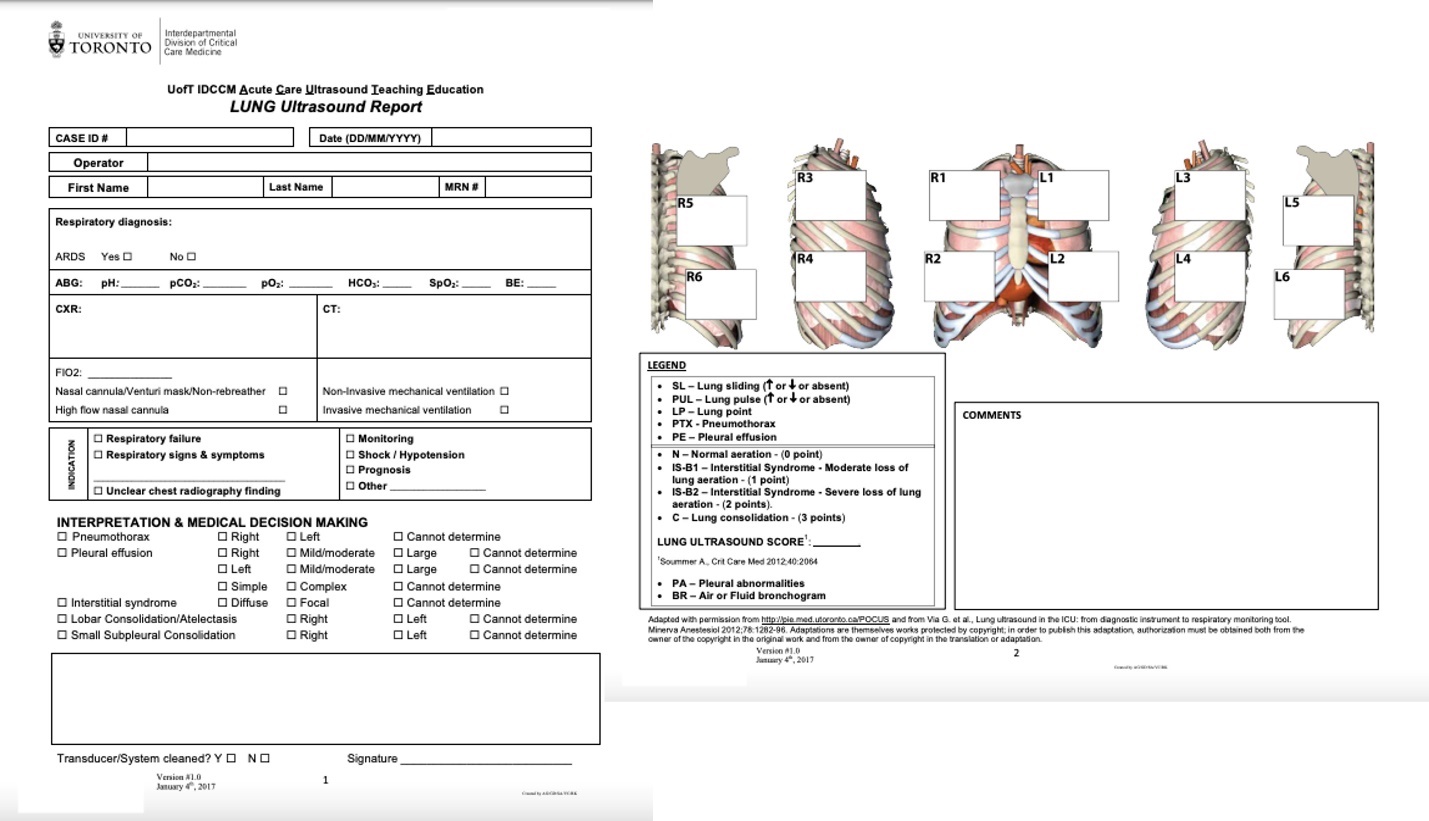
**

**SUPPLEMENTAL FIGURE 13.** A sample LUS report. Reproduced with permission from Dr. Goffi^28^.
