# Supplementary material for: Nuts and bolts of lung ultrasound: utility, scanning techniques, protocols, and findings in common pathologies
Source: Crit Care. 2024 Oct 7;28:328. doi: 10.1186/s13054-024-05102-y (PMC11460009; doi:10.1186/s13054-024-05102-y)
Supplement: Supplementary file 6 — Additional file 6 [file 13054_2024_5102_MOESM6_ESM.docx]

| **Major Society Organization(s)** | **Available Dedicated Lung Ultrasound Program for Accreditation, Certification, or Competency** | **General Ultrasound Programs that include Lung Ultrasound for Accreditation, Certification or Competency** | | |
| --- | --- | --- | --- | --- |
|  |  | **Programs** | **Target Audience** | **Program Recommendations and Requirements specific to Lung Ultrasound** |
| American College of Chest Physicians ^156,159^ | None | Point-of-Care Ultrasound Certificate of Completion ^156^  Critical Care Ultrasonography Certificate of Completion ^159^ | Hospitalists (internal medicine, family medicine), Advanced practice providers, Physician Assistants  All health care providers, relevant to clinicians caring for acutely hospitalized patients | - Complete Online e-learning modules  - Attend in-person ultrasound courses  - Complete online portfolio (Lung specific requirements include 5 studies with the following views per study: Normal sliding with A-lines, Consolidation, Pleural effusion, B-lines)  - Pass comprehensive skills and knowledge assessments  - Complete online e-learning modules  - Attend in-person ultrasound courses  - Complete online portfolio (Lung specific requirements include 4 images of pleural Effusion, lung sliding, and consolidation)  - Pass comprehensive final assessment |
| American College of Emergency Physicians ^158^ | None | Residency or Practice Based Pathway for Emergency Ultrasound | Emergency medicine residents or attendings | Complete of ultrasound course/didactics or a 1-2 week preceptorship, followed by a period of supervision during clinical application; recommend completion of 25-50 exams in each domain |
| American Society of Anesthesiology ^157^ | None | Diagnostic POCUS Certificate Program | Physician anesthesiologists | - Complete quality improvement action plan  - 12 hour of ultrasound training (of which an available basic lung ultrasound course is 2 CME hours)  - 160 unique case image interpretation training (of which 20 are lung cases), 140 image acquisition portfolio (of which 30 are lung ultrasound exams)  - Completion of final exam |
| Society of Point-of-Care Ultrasound ^162^ | None | POCUS Practice Guidelines for Competency | Healthcare providers performing ultrasound | Didactics and hands-on training,25-50 supervised exams for each domain (with 5% demonstrating pathology), followed by ongoing quality assurance |
| Society of Critical Care Medicine (SCCM) ^161,164^ | None | Critical Care Ultrasound (CCUS) with Focused Ultrasound Pathway | Critical care physician | - At least 20 didactic hours  -Interpret a minimum of 140 diagnostic (of which 30 are lung related) and 30 procedural (of which 5 are lung related) ultrasound exams  - Perform a minimum of 90 diagnostic (of which 20 are lung related) and 30 procedural (of which 5 are lung related) |
| National Board of Echocardiography (NBE) ^163^ | None | Advanced Critical Care Echocardiography Certification Pathway | Critical care physician | Passed CCEeXAM or ASCeXAM, board certification, critical care training (supervised training pathway) or practicing with 20 hours of CME credit (practice experienced pathway), performed and interpreted 150 medically necessary critical care transthoracic echocardiogram exams (must include cardiac ultrasound, no minimum lung ultrasound studies) |
| British Society of Echocardiography/ Intensive Care Society ^165^ | None | Adult Critical Care Echocardiography Accreditation | Critical care physician | - Pass written exam  - Practical scanning assessment (10 different echocardiographic imaging views within 20 minutes on real-life model or simulator)  - Viva (ie. oral) assessment of five separate patient case studies  - 250 case logbook (at least 10 cases for refractory hypoxemia/difficulty weaning) |
| European Society of Intensive Care Medicine (ESICM) ^160^ | None | European Diploma in Advanced Critical Care Echocardiography (EDEC) | Critical Care Physicians | - Pass a written and practical examination  - Complete advanced course at ESICM annual congress and online modules  - Supervised case logbook of a minimum of 35 TEE and 100 TTE clinical cases (no minimum lung ultrasound studies) |
| European Respiratory Society ^166^ | None | Thoracic ultrasound training program | Health Care Professionals | Three components: an online theoretical module (Part 1), a practical course (Part 2) that includes 2 on-line and 2 on-site courses and an examination (Part 3), which includes an (OSCE) conducted annually at the European Respiratory Society Congress |

**SUPPLEMENTAL TABLE 1.** Major Societies’ Available Dedicated Lung Ultrasound Programs and General Ultrasound Programs that include Lung Ultrasound for Accreditation, Certification, or Competency.
